# Evaluation of HIV protease and nucleoside reverse transcriptase inhibitors on proliferation, necrosis, apoptosis in intestinal epithelial cells and electrolyte and water transport and epithelial barrier function in mice

**DOI:** 10.1186/1471-230X-10-90

**Published:** 2010-08-11

**Authors:** Manuel B Braga Neto, Carolina V Aguiar, Jamilly G Maciel, Bruna MC Oliveira, Jesus E Sevilleja, Reinaldo B Oriá, Gerly AC Brito, Cirle A Warren, Richard L Guerrant, Aldo AM Lima

**Affiliations:** 1 Institute of Biomedicine and Clinical Research Unit-University Hospital, Federal University of Ceará, Fortaleza, Brazil; 2 Center for Global Health, Division of Infectious Diseases and International Health, University of Virginia, Charlottesville, USA; 3 Department of Morphology, Federal University of Ceará, Fortaleza, Brazil; 4 Department of Physiology and Pharmacology, Federal University of Ceará, Fortaleza, Brazil

## Abstract

**Background:**

Protease inhibitors (PI's) and reverse transcriptase drugs are important components of highly active antiretroviral therapy (HAART) for treating human acquired immunodeficiency syndrome (AIDS). Long-term clinical therapeutic efficacy and treatment compliance of these agents have been limited by undesirable side-effects, such as diarrhea. This study aims to investigate the effects of selected antiretroviral agents on intestinal histopathology and function *in vivo *and on cell proliferation and death *in vitro*.

**Methods:**

Selected antiretroviral drugs were given orally over 7 days, to Swiss mice, as follows: 100 mg/kg of nelfinavir (NFV), indinavir (IDV), didanosine (DDI) or 50 mg/kg of zidovudine (AZT). Intestinal permeability measured by lactulose and mannitol assays; net water and electrolyte transport, in perfused intestinal segments; and small intestinal morphology and cell apoptosis were assessed in treated and control mice. *In vitro *cell proliferation was evaluated using the WST-1 reagent and apoptosis and necrosis by flow cytometry analysis.

**Results:**

NFV, IDV, AZT and DDI caused significant reductions in duodenal and in jejunal villus length (p < 0.05). IDV and AZT increased crypt depth in the duodenum and AZT increased crypt depth in the jejunum. NFV, AZT and DDI significantly decreased ileal crypt depth. All selected antiretroviral drugs significantly increased net water secretion and electrolyte secretion, except for DDI, which did not alter water or chloride secretion. Additionally, only NFV significantly increased mannitol and lactulose absorption. NFV and IDV caused a significant reduction in cell proliferation *in vitro *at both 24 h and 48 h. DDI and AZT did not alter cell proliferation. There was a significant increase in apoptosis rates in IEC-6 cells after 24 h with 70 ug/mL of NFV (control: 4.7% vs NFV: 22%) while IDV, AZT and DDI did not show any significant changes in apoptosis compared to the control group. In jejunal sections, IDV and NFV significantly increased the number of TUNEL positive cells.

**Conclusion:**

The PI's, NFV and IDV, increased cell apoptosis *in vivo*, water and electrolyte secretion and intestinal permeability and decreased villus length and cell proliferation. NFV was the only drug tested that increased cell apoptosis *in vitro*. The nucleoside reverse transcriptase inhibitors, AZT and DDI, did not affect cell apoptosis or proliferation. These findings may partly explain the intestinal side-effects associated with PI's.

## Background

Protease inhibitors (PI's) and reverse transcriptase drugs (RTs) are important components of highly active antiretroviral therapy (HAART) for treating human acquired immunodeficiency syndrome (AIDS) and have significantly changed the natural history of AIDS, increasing life expectancy and quality of life [1,2]. These drugs are known to reduce human immunodeficiency virus (HIV) load and increase circulating CD4 T cells, thus resulting in fewer gastrointestinal conditions, ultimately reducing hospitalization, and enhancing life expectancy in HIV-infected individuals [3,4]. However, long-term clinical therapeutic efficacy and treatment compliance of these agents have been limited by undesirable side-effects, such as diarrhea, most commonly seen with ritonavir-boosted PI's, and enteric infections, which have been reported to occur in up to 62% of patients [4-6].

Although life-saving benefits of HAART are well-known, antiretroviral intestinal side-effects have not been explored in animal models and the mechanisms of HAART-induced intestinal epithelial damage *in vitro *are still poorly understood. There is a scarcity of *in vivo *and *in vitro *studies that evaluate the effects of these agents on intestinal barrier function. Bode *et al*, 2005, reported decreased transepithelial resistance in HT-29 monolayers after treatment with PI's, accompanied by massive cell apoptosis but not necrosis [7]. Since these initial findings, several studies have been conducted both *in vitro *and *in vivo*, suggesting the potential benefits of HIV PI's in chemotherapy [8]. Nelfinavir has been demonstrated to induce endoplasmic reticulum (ER) stress, autophagy and apoptosis both *in vivo *and *in vitro *and the authors have, therefore, suggested it could be repositioned as a chemotherapy agent [9]. Another study demonstrated that PI's induce secretory diarrhea, by potentiating muscarinic chloride secretion in T84 cells through amplification and prolongation of an apical membrane Ca^2+^-dependent chloride conductance [10].

Our group has demonstrated that intestinal epithelial barrier breakdown due to enteric infections and diarrhea might lead to antiretroviral drug malabsorption and increased drug resistance [11]. Improvements of gastrointestinal symptoms and antiretroviral drug levels were found with oral glutamine derivatives in a randomized clinical trial enrolling hospitalized AIDS patients after seven days of intervention [12]. However, it is of great importance to further understand the mechanisms involved in this process in order to optimize possible future interventions that can lead to a decrease in side effects such as diarrhea.

The intestinal epithelial barrier is composed of an extremely dynamic cell population, which behaves differently during intestinal adaptation following mucosal injury [13]. This epithelial lining is renewed with a highly active cell turn over by means of their surrounding crypts, from where stem cells migrate and differentiate towards the villus tip [14-17]. This current study assessed changes in the intestinal barrier function from selected anti-retroviral drugs in mice and in intestinal epithelial cells, thus shedding light on specific drug-host interactions and intestinal side effects of targeted antiretroviral therapy.

## Methods

### Reagents and drugs

Mellibiose and chemicals for enteric perfusion, including NaCl, KCl, CaCl_2_, NaHCO_3_, and NaH_2_PO_4_, and sodium citrate were obtained from Sigma-Aldrich, St. Louis, MO. Lactulose and mannitol were purchased from Luitpold Produtos Farmacêuticos Ltda., Barueri, SP, and from Henrifarma Produtos Químicos e Farmacêuticos, São Paulo, SP, Brazil, respectively. Nelfinavir (NFV), indinavir (IDV), zidovudine (AZT), and didanosine (DDI) were kindly granted by the Infectious Diseases' State Hospital São José, Fortaleza, CE, Brazil, solely for the purpose of rodent experiments. Tetrazolium salt WST-1 reagent was obtained from Roche (Mannheim, Germany). Mitomycin C was obtained from Roche (Mannheim, Germany). Annexin V Apoalert kit with Binding buffer was obtained from BD Biosciences (Clonetech, Palo Alto, CA). ApopTag Plus Peroxidase In Situ Detection Kit (Serologicals Corp., Norcross, GA). AZT, DDI, IDV and NFV used for cell culture compounds were obtained through the AIDS Research and Reference Reagent Program, Division of AIDS, NIAID, NIH.

### Animals

Male Swiss mice weighing 30 to 40 g were obtained from the Clinical Research Unit & Institute of Biomedicine animal facility (Federal University of Ceara), and were housed in temperature-controlled rooms prior to animal experiments. All animals received standard mice chow diet and water *ad libitum*. Surgical procedures, animal handling and treatment were reviewed and approved by the Animal Care and Use Committee at the Federal University of Ceara, according to the Brazilian College for Animal Experimentation guidelines (COBEA).

### Treatment regimen

Animals were weighed and randomized into different treatment groups. NFV, IDV, AZT, and DDI were diluted in sterile phosphate buffer (PBS), as vehicle, and a control group treated only with PBS. Each group consisted of six or more animals. NFV (100 mg/kg), IDV (100 mg/kg), AZT (50 mg/kg) and DDI (100 mg/kg) or PBS, were given orally by gavage on days 1 through 7. In order to find an evaluable regimen for testing, we conducted a pilot study to evaluate survival rates using the following concentrations: NFV (100 and 300 mg/kg); IDV (300 mg/kg); AZT (100 and 300 mg/kg) and DDI (150 and 100 mg/kg). Doses and the experimental course (one week-treatment) were defined based on the survival data. All animals were weighed and clinically examined daily for diarrhea until the study end point. Data on nutritional intake was not collected during the time of the experiment. Therefore, we cannot exclude differences in nutritional intake between the groups.

### Histology and Intestinal Morphometry

Mice were sacrificed by a lethal injection of a euthanasia solution containing chloral hydrate (250 mg/kg, i.p), under anesthesia, on day 8. Immediately after euthanasia, 0.5 cm-samples were harvested from different intestinal segments, as follows: duodenum, jejunum, and ileum, based on anatomical hallmarks. Tissue specimens were fixed in 10% neutral buffered formalin, and dehydrated for 12 h. On the following day, specimens were cut with a razor blade and then stored in 70% ethanol for paraffin embedding. 5 um-thick cross-sections were prepared for hematoxylin-eosin staining (HE). Crypt depth and villus height were measured from HE stained slides on a light microscope equipped with a digital camera, and a computer-aided image capture system. Villus height was measured from the tip to the villus-crypt junction. The crypt depth was measured from the villus-crypt junction to the crypt bottom. At least 10 clear longitudinal sections of the villi and the crypts were selected randomly from each sample, measured with an eyepiece ruler and averaged after proper calibration. All morphometric analyses were conducted blindly regarding experimental groups and diarrheal outcomes. Morphologic analyses were carried out by only one investigator in a blinded manner; hence inter or intra-individual variability was not assessed.

### In vivo Analysis for Cell Death

Analysis of apoptosis or necrosis was performed using ApopTag Plus Peroxidase In Situ Detection Kit (Serologicals Corp., Norcross, GA) for TUNEL (terminal deoxynucleotidyltransferase-mediated dUTP-biotin nick end labeling). The ApopTag Plus Peroxidase. In Situ Detection Kit distinguishes apoptosis from necrosis by specifically detecting DNA cleavage and chromatin condensation associated with apoptosis. However, there may be some instances where cells exhibiting necrotic morphology my stain lightly or in rare instance, DNA fragmentation can be absent or incomplete in induced apoptosis. Thus, the results were presented as TUNEL positive cells as recommended [18]. Pariffin-embedded intestinal tissue samples sections were hydrated and incubated with 20 μg/ml of proteinase K (Sigma, New York) for 15 minutes at room temperature (RT). Endogenous peroxidase was blocked by treatment with 3% (wt/vol) hydrogen peroxide in PBS for 5 minutes at RT. Slides were then washed with PBS and sections were incubated in a humidified chamber at 37°C for 1 h with TdT buffer containing TdT enzyme and reaction buffer. Afterwards, samples were incubated for 10 min at RT with a stop/wash buffer and then incubated in a humidified chamber for 30 min with anti-digoxigenin-peroxidase conjugate at RT. Samples were then washed several times in PBS, the slides were covered with peroxidase substrate to develop color and then wash in three changes of distilled H_2_O and counterstained in 0.5% (vol/vol) methyl green for 10 minutes at RT. Cell apoptosis was measured by counting under a light microscope the number TUNEL positive cells, which represent apoptotic cells and possibly some necrotic cells. At least 10 randomly selected sections from each sample were counted and averaged.

### Intestinal Absorption and Permeability

The lactulose/mannitol ratio (L:M) was used in this study as the primary parameter to evaluate the integrity of the intestinal barrier function. Mannitol, a monosaccharide, is considered a biological marker of total intestinal absorptive area, since it is absorbed transcellularly. In contrast, lactulose, a disaccharide, is a marker of mucosal damage, since it is only absorbed paracellularly. Animals were fed with a low carbohydrate-chow diet on days 5, 6 and 7 of the treatment regimen. On day 7, mice were fasted overnight (8-12 h) and placed in metabolic cages (3 animals/cage). On day 8, 0.25 ml of a solution containing lactulose (20 mg/mL) and mannitol (50 mg/mL) was given to the experimental mice by gavage. After 1 h the animals regained access to food and water *ad libitum*. The urine was collected during the next 24 hours in a flask and mixed with a 25 uL-solution containing chlorhexidine (40 mg/mL). Total urine volumes were collected, aliquoted to 50 μl and stored at -20°C for further analyses. Each urine sample (50 *u*l) was mixed with 50 uL of a solution containing mellibiose (3.6 mM) and diluted in 2.9 ml of doubled-distilled and deionized water. After centrifugation and filtration via a Millipore membrane (0.22 *u*m), a 50 uL-filtered urine solution was employed for sugar determination using high-performance liquid chromatography (HPLC) with pulsed amperometric detection, as previously described [19]. Urinary recovery of both lactulose and mannitol was calculated as a percentage of the dose ingested.

### Intestinal Net Fluid and Electrolyte Transport

On day 7, experimental mice treated either with NFV, IDV, AZT, DDI or PBS, were fasted for 12 h, with free access to water *ad libitum*. Animals were submitted to intestinal perfusion to evaluate net fluid and electrolyte transport. After ketamine (35 mg/kg, i.m.) and xylazine (5 mg/kg, i.m.) anesthesia, a median 3- to 5-cm laparotomy was performed for visualization of the small intestine. An approximately 15-cm ileal segment was selected and washed with 1 ml of phosphate-buffered saline (pH 7.4), and the proximal and distal ends were ligated by means of polyvinyl cannulas for tissue perfusion. Polyvinyl cannulas (internal diameter 0.08 cm.; outer diameter 0.26 cm; Cole-Parmer Instrument Company, Vernon Hills, IL) were inserted approximately 5 cm distal to the ligament of Treitz and 5 cm proximal to the ileocecal valve (internal diameter, 0.085 in.; outer diameter 0.128 in.; Becton Dickinson, Sparks, MD). Ringer's solutions were pre-warmed to 37°C, maintained at pH 7.4, and introduced through the proximal cannula with the aid of a motorized pump (Masterflex C/L Pump System, Model 77120-62; Cole-Parmer Instrument Co.). Perfusion was maintained at the slow rate of 0.16 ml/min throughout the experiment. Samples were taken every 15 min throughout the 75-min study period for electrolyte and osmolarity measurements, as well as for determination of phenolsulfonphthalein (PSP) concentration. At the end of the perfusion, the animals were sacrificed, and the dry weight (after desiccation at 90°C for 72 h) of the intestinal segment was used to calculate the perfusate flow and net water and electrolyte transport.

### Biochemical Analyses

PSP (50 *u*g/ml) was used as a non-absorbable marker for sodium, potassium, chloride and water net flux calculation. PSP was measured spectrophotometrically (Spectrophotometer Model C382; Microsonal S.A, São Paulo, SP, Brazil) according to the method developed by Schedl and Clifton [20]. Sodium and potassium concentrations in the perfusate were measured by flame photometry (Flame Photometer Model 443; Instrumentation Laboratory, Lexington, MA). The colorimetric method for chloride detection (Labtest Bio. Diagnostics, Belo Horizonte, MG, Brazil) was used according to the manufacturer's instructions. The osmolarity of the perfusion samples was measured with a vapor pressure osmometer (Model 5100C; Wescor, Logan, UT).

### Cell culture

Rat intestinal jejunal crypt cells (IEC-6, passages 8-14) were purchased from American Type Culture Collection (Rockville, MD) and were cultured at 37°C in a 5% CO2 incubator. The maintenance cell media was Dulbecco's Modified Eagle Media (DMEM; Gibco BRL, Grand Island, NY) supplemented with 5% Fetal Bovine Serum (FBS), 5 mg bovine insulin, 50 ug/ml of penicillin/streptomycin (DMEM; Gibco BRL, Grand Island, NY) and a final concentration of 1 mM of sodium pyruvate. The media was changed thrice a week, according to standard culture protocols. The cultured cells were trypsinized with 0.05% EDTA trypsin when 90-95% confluence was achieved.

### Cell proliferation

Cell proliferation was measured indirectly using the tetrazolium salt WST-1 (4-[3-(4-iodophenyl)-2H-5-tetrazolio]-1-3-benzene disulfonate), according to the manufacturer recommendations. A 96-well plate was seeded with IEC-6 cells in a total concentration of 4 × 10^3 ^cells/well in 100 uL of DMEM media. Cells were allowed to attach for 48 hours, when the wells were washed with 100 uL of DMEM media. The cells were then incubated for 24 and 48 h with either DMEM media or DMEM media incubated with NFV (7 ug/mL, 10 ug/mL, 70 ug/mL and 100 ug/mL), DDI (5 ug/mL, 10 ug/mL, 50 ug/mL and 100 ug/mL), IDV (5 ug/mL, 10 ug/mL, 50 ug/mL and 100 ug/mL) and AZT (5 ug/mL, 10 ug/mL, 50 ug/ml and 100 ug/mL). After 24 and 48 hours, wells were incubated for 4 hours with 10 uL of the tetrazolium salt and the absorbance was measured using an ELISA microplate reader at 450 nm (reference range 420-480 nm). Tetrazolium salts are cleaved to formazan by mitochondrial enzymes in viable cells. Enhancement of the number of viable cells will result in an increase of the amount of the formazan dye, which is detectable by the ELISA reader. Therefore, this model indirectly measures cell proliferation in a time-dependent-manner. The experiments were carried out separately with individual control groups for each experiment, using different cell passages. Therefore, the analyses were done comparing values within each individual experiment.

### Flow Cytometry for Apoptosis and Necrosis

Apoptosis and necrosis were measured by flow cytometry analyses using the ApoAlert annexin V kit. Annexin V is a molecule that binds to phosphatidylserine (PS) and when conjugated to a fluorochrome detects apoptotic cells expressing PS on the reversed membrane surface. For this protocol, propidium iodide was also used to detect necrotic and late apoptotic cells, which express propidium iodide inside the membrane. The cells were seeded on 12-well plates in a concentration of 5 × 10^5^cells/well. These cells were allowed to attach on the plate surface for 24 hours. Afterwards, cells were washed with DMEM media and incubated with NFV (70 u/mL), IDV (100 ug/mL), DDI (100 ug/mL), AZT (100 ug/mL). After 24 h of incubation, cells were trypsinized, centrifuged, and washed with serum-containing media, before incubation with annexin V. Cells were counted and diluted to 10^5^-10^6 ^cells and rinsed with 1× Binding Buffer, and re-suspended in 200 uL of Binding Buffer. 5 uL of annexin V and 10 uL of propidium iodide were added and incubated for 5-15 min in the dark. The samples were then processed at the University of Virginia's Flow Cytometry Core, using a FACS Calibur dual laser (Becton Dickinson).

### Statistical Analyses

Results are expressed as mean ± standard error (SEM), as generated by GraphPad Prism version 4.0 (GraphPad software, San Diego, CA). The differences between the experimental groups were compared by one-way ANOVA, corrected by Bonferroni's multiple comparison tests.

## Results

### 1) In vivo experiments

#### Survival rate and body weight

**Figure 1:**
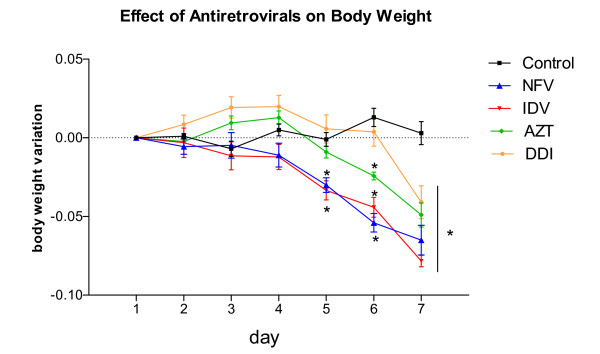
**Effect of NFV (100 mg/kg), IDV (100 mg/kg), AZT (50 mg/kg) and DDI (100 mg/kg) on body weight in mice**. Each animal was weighed daily throughout the experiment. The results are expressed as mean ± SEM of at least six mice per group. *statistical significance (*P *< 0.05) compared to the PBS control group by ANOVA, Bonferroni's multiple test.

In order to define possible antiretroviral toxicity in our murine model, we have tested preliminarily different concentrations of antiretroviral drugs for 5 consecutive days. The tested concentrations are as follows: NFV (100 and 300 mg/kg); IDV (300 mg/kg); AZT (100 and 300 mg/kg) and DDI (150 and 100 mg/kg). The only group that presented significant toxicity was AZT, featured by mucous diarrhea, wasting, and death at the dose of 100 mg/kg (2/8 = 25%) and 300 mg/kg (4/8 = 50%). Therefore, we decided to decrease AZT concentration to 50 mg/kg. The minimum effective and safe dose of the antiretroviral agents was chosen for the remainder of the study. In order to assure a longer period of treatment, we increased the duration of antiretroviral therapy to seven days. All animals (at least n = 6 per group) survived throughout the experimental course. NFV and IDV groups showed significant weight reductions as early as five treatment days. Seven days following treatment all antiretroviral groups showed significant weight decrements as compared to the untreated PBS control (p < 0.001), as seen in Figure 1.

#### Effect of Antiretrovirals on Intestinal Villi Length, Crypt Depth and Cell Apoptosis

**Figure 2:**
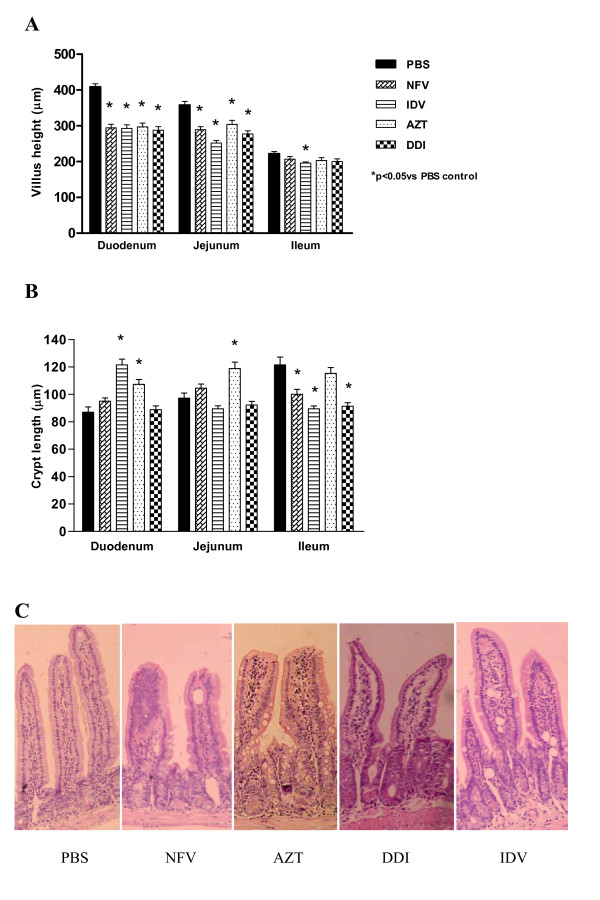
**Villus height (A) and crypt depth (B) after 7 days of treatment with either nelfinavir (NFV, 100 mg/kg), indinavir (IDV, 100 mg/kg), zidovudine (AZT, 50 mg/kg) and didanosine (DDI, 100 mg/kg) or PBS**. For each animal, a total of 10 villi and 10 crypts were measured for each segment of duodenum, jejunum, and ileum with a micrometer. The results are expressed as mean ± SEM of six mice per group. *statistical significance (*P *< 0.05) compared to the PBS control group by ANOVA, Bonferroni's multiple test. **(C) **Illustrative pictures of the effects of CONTROL (PBS), NFV (100 mg/kg), DDI (100 mg/kg), IDV (100 mg/kg), AZT (50 mg/kg) on the intestinal jejunal tissue, showing decreased villi length caused by all antiretrovirals and increased crypt depth caused by AZT.

As shown in Figure 2A, antiretroviral drugs strikingly blunted duodenal villus height at day 8, as compared to the unchallenged group treated with PBS (p < 0.001). Proximal and mid-segments of the small intestine were most affected. IDV was the only drug tested that caused a significant decrease in ileal villus height. IDV significantly reduced villus height throughout the small intestinal segments, having a significant blunting effect in both the duodenum and ileum (p < 0.05). As seen in Figure 2B, this morphological villus atrophy due to IDV was accompanied by increased crypt depth in the duodenum, and decreased crypt depth in the ileum. Most of the antiretroviral drugs tested (NFV, IDV and DDI) decreased the crypt depth in the ileal mucosa. A similar trend was seen with AZT, albeit not reaching statistical significance. Interestingly, only AZT induced crypt hyperplasia in both the duodenum and jejunum, in contrast to the other antiretroviral drugs, except IDV which only caused crypt hyperplasia in the duodenum. Figure 2C illustrates the effects of both protease inhibitors and reverse transcriptase on the intestinal villous length and crypt depth.

**Figure 3:**
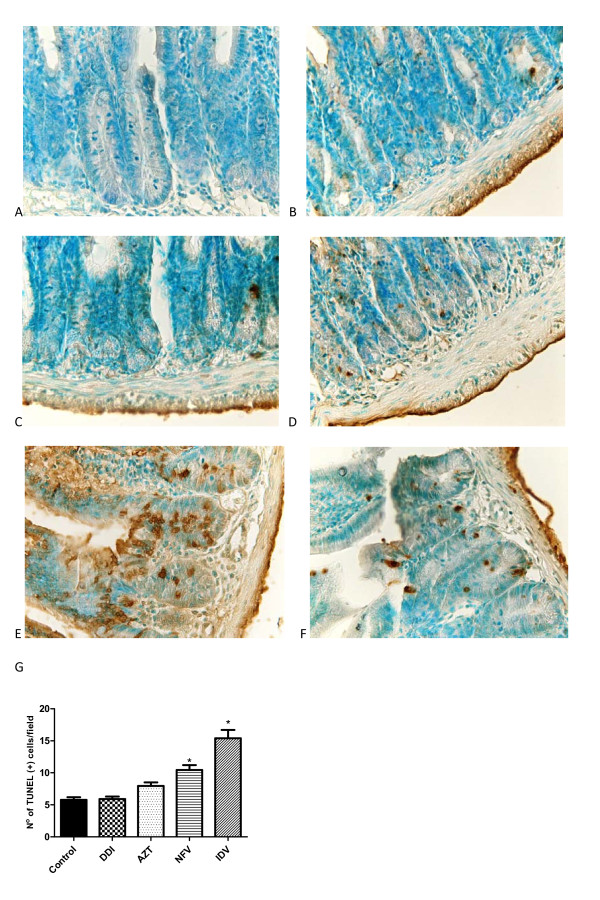
**Illustrative pictures of the effects of antiretrovirals on cell death detected by TUNEL method (A) Negative-control (B) PBS control, (C) DDI 100 mg/kg, (D) AZT 50 mg/kg, (E) IDV 100 mg/kg and (F) NFV 100 mg/kg on cell death, indicated by the brown staining of the cells detected by the TUNEL method in the jejunal tissue, (G) Effects of the antiretrovirals on cell death, expressed as average of number of TUNEL-positive cells**. P < 0.05 when compared to control group (B) by one-way ANOVA and Bonferroni's test.

Both NFV and IDV increased the number of TUNEL positive cells (apoptosis and possibly necrosis) significantly by 89.6% and 180% compared to controls (cell death is indicated by the brown staining of the cells detected by the TUNEL method; Figure 3). AZT and DDI did not cause a significant increase in the number of TUNEL positive cells.

#### Intestinal Electrolyte and Water Absorption

**Figure 4:**
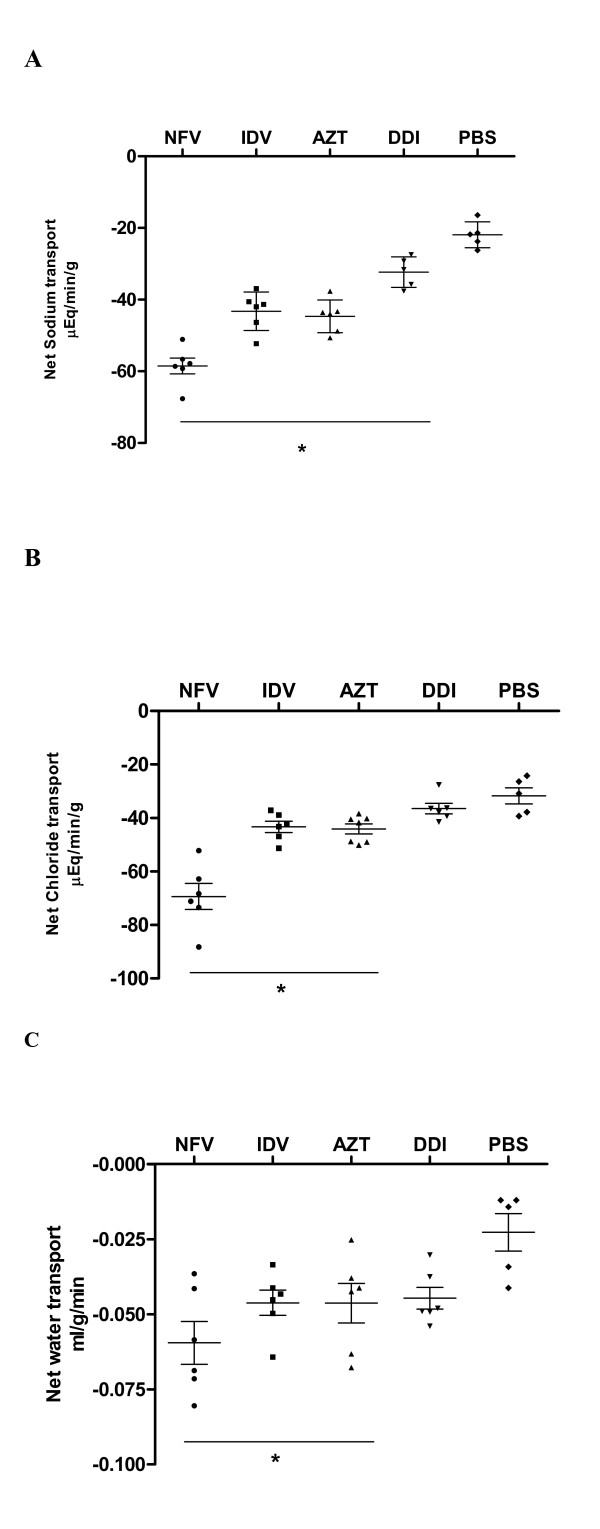
**Intestinal transport of fluid and electrolytes (mEq/min/g) in experimental mice (n = at least 5 per group) after 7 days of treatment with either nelfinavir (NFV, 100 mg/kg), indinavir (IDV, 100 mg/kg), zidovudine (AZT, 50 mg/kg) and didanosine (DDI, 100 mg/kg) or PBS**. Mice undergoing protease and reverse transcriptase inhibitors were submitted to intestinal perfusion (60 min) with ringer lactate. The results are expressed as mean ± SEM of at least 5 mice per group. *statistical significance (*P *< 0.05) compared to the PBS control group by ANOVA, Bonferroni's multiple test. A: sodium; B: chloride and C: fluid transport.

NFV treatment caused significant increases in sodium (167%), chloride (118.7%), and water (260%) secretion when compared to controls. Likewise, IDV caused significant increases, in sodium (97.5%), chloride (36.5%) and water (95.7%) secretion. Treatment with AZT also increased sodium (102.9%), chloride (36.6%) and water (117.4%) secretion. Treatment with DDI increased only sodium secretion (47.67%). (Figure 4)

#### Intestinal Absorption and Permeability

**Figure 5:**
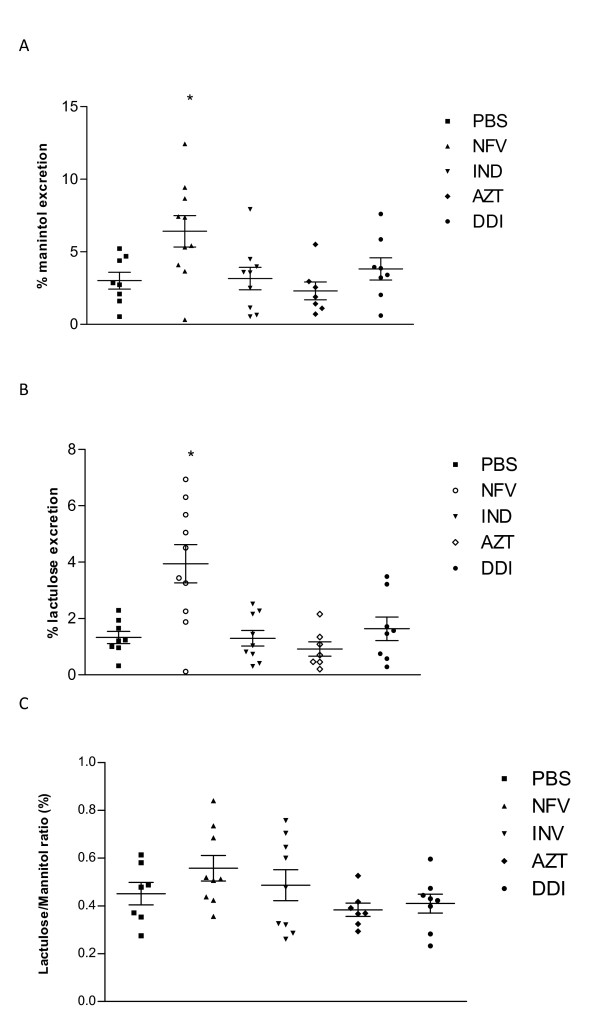
**Lactulose and mannitol excretion (%) in experimental mice (n = at least7 per group) following 7 days of treatment with either nelfinavir (NFV, 100 mg/kg), indinavir (IDV, 100 mg/kg), zidovudine (AZT, 50 mg/kg) and didanosine (DDI, 100 mg/kg) or PBS**. *statistical significance (*P *< 0:05) compared to the PBS control group by ANOVA and Bonferroni's multiple test.

NFV treatment increased lactulose and mannitol excretion significantly (p < 0.05). Lactulose excretion increased from 1.32 ± 0.24% to 3.94 ± 0.68%. However, mannitol excretion was also significantly increased from 3.014 ± 0.6% to 6.41 ± 1,08% after NFV chronic administration; hence there was no difference in the L/M ratio, when compared to the untreated control. Neither IDV, AZT nor DDI treatment altered the excretion of mannitol, lactulose or the L/M ratio (Figure 5).

### 2) In vitro experiments

#### Cell proliferation

**Figure 6:**
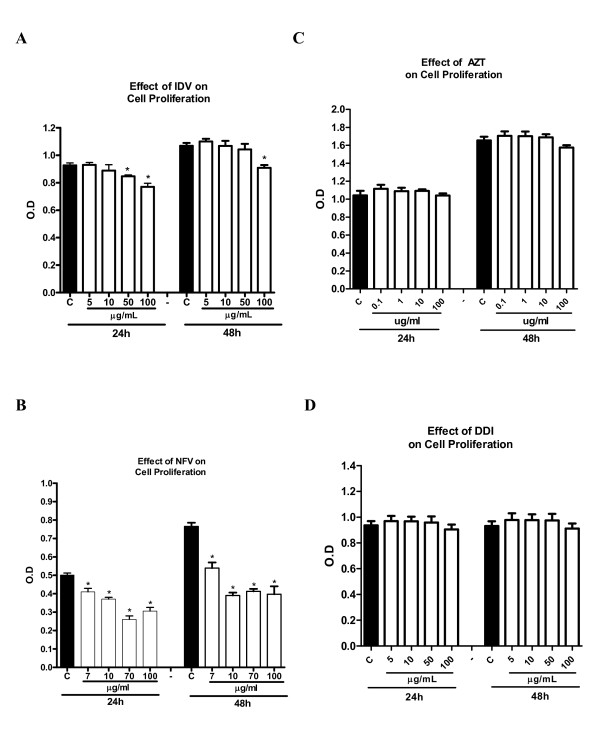
**Effect of four different concentrations of nelfinavir (A), indinavir (B), zidovudine (C) and didanosine (D) on intestinal cell proliferation after 24 h and 48 h of exposure by detected absorbance using an ELISA microplate reader at 420 nm in 96 well plates seeded with IEC-6 cells**. After an exposure time of 24 h and 48 h, with the antiretroviral drugs, 10 uL of the tetrazolium salt was added to each well, and absorbance was measured after 4 h of incubation. * P < 0.05 when compared to control group (C) incubated in standard DMEM media using one-way ANOVA and Bonferroni's test.

The protease inhibitors, NFV and IDV, caused a significant reduction in IEC-6 cell proliferation at both 24 h and 48 h. NFV significantly inhibited cell proliferation at doses of 7, 10, 70 and 100 ug/mL at 24 h (reduction of 18.1%, 26.1%, 48.1% and 39.1%, respectively; p < 0.05; Figure 6A) and at 48 h (reduction of 29.5%, 49%, 46%, 48.2%, respectively; p < 0.05; Figure 6A). IDV at doses of 50 ug/mL and 100 ug/mL significantly inhibited cell proliferation by 8.6% and 17%, respectively, at 48 h and by 15% at the dose of 100 ug/mL (p < 0.05; Figure 6B). Slight (4.7%) but not significant inhibition of cell proliferation was seen with AZT at the dose of 100 ug/mL at 48 h, with inhibition of 4.7%, which was not statistically significant (Figure 6C; P > 0.05). Similarly, DDI exposure did not show any significant inhibition of cell proliferation at either 24 h or 48 h (Figure 6D).

#### Apoptosis and Necrosis

**Figure 7:**
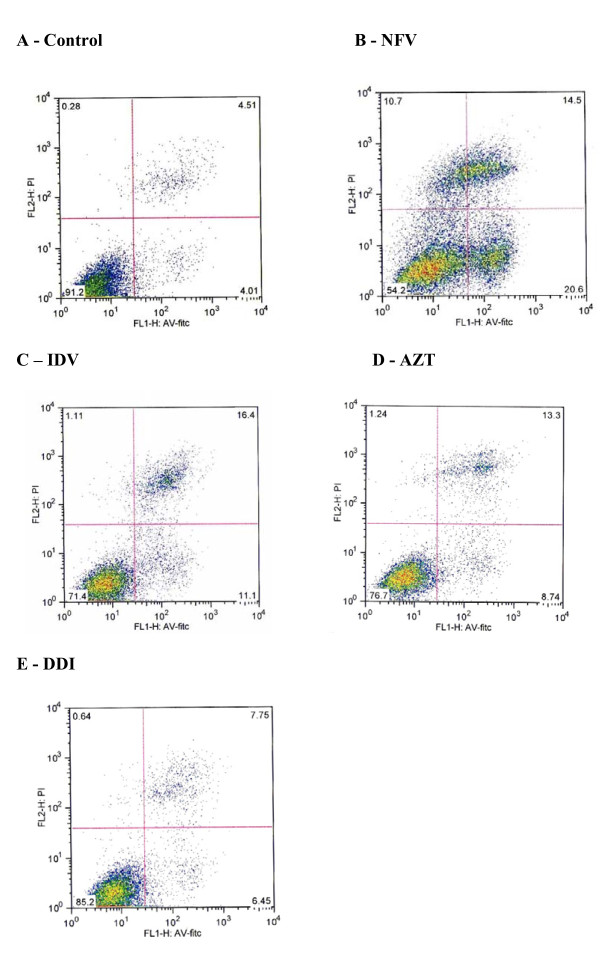
**Rates of apoptosis and necrosis of control group (A), of 70 ug/mL of nelfinavir (B), 100 ug/mL of indinavir (C), zidovudine (D) and didanosine (E) on intestinal epithelial IEC-6 cell apoptosis and necrosis after 24 h of exposure**. Cells were stained with FITC-conjugated annexin V and propidium iodide and analyzed by flow cytometry. Results are shown as density plots with propidium iodide vs. annexin V-FITC. Viable cells have low annexin-V-FITC and low propidium iodide staining (lower-left quadrant), apoptotic cells have high annexin V-FITC and low propidium iodide staining (lower-right quadrant), and necrotic cells have high propidium iodide and annexin V-FITC staining (upper-right quadrant).

**Figure 8:**
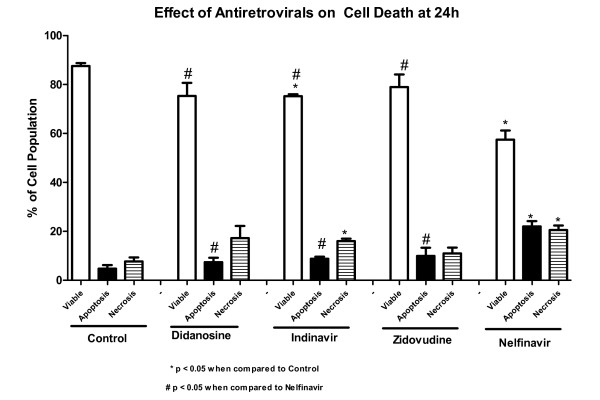
**Percentage of cell apoptosis, necrosis, and viable cells of the untreated control group, of 70 ug/mL of NFV, 100 ug/mL of IDV, AZT and DDI on intestinal epithelial IEC-6 cell apoptosis and necrosis after 24 h of exposure**. * P < 0.05 when compared to control group (C) incubated in standard DMEM media and # P < 0.05 when compared to the nelfinavir group by one-way ANOVA and Bonferroni's test.

Triplicate flow cytometry analyses demonstrated a significant increase in apoptosis in IEC-6 cells after 24 h incubation with 70 ug/mL of NFV (Control: 4.7% *vs *NFV: 22%; P = 0.0001; Figures 7 and 8). Slight, but insignificant increases in IEC-6 cells apoptosis were seen after 24 h of exposure to 100 ug/mL IDV, 100 ug/mL AZT and 100 ug/mL DDI (Control: 4.7% *vs *IDV: 8.8%; AZT: 9.9%; DDI: 7.4%; P: 0.29). There was a significant increase in IEC-6 cell necrosis rates in IEC-6 after 100 ug/mL IDV or 70 ug/mL NFV(Control: 7.7% vs 16.0%; P < 0.05 and 20.54%; P < 0.05), as shown in Figures 7 and 8. There were slight increases in necrosis with at 100 ug/mL AZT and 100 ug/mL DDI that were not statistically significant. Additionally, the apoptosis rates were significantly higher with NFV than with IDV, AZT or DDI apoptosis rates (P < 0.05 by one-way ANOVA test).

## Discussion

Gastrointestinal side effects of HAART are usually well tolerated and do not contribute to significant treatment discontinuation. However, diarrhea of moderate to severe intensity can occur in patients receiving multi-drug therapy [21-23]. The mechanisms underlying antiretroviral-induced diarrhea are unclear. The intestinal epithelium acts as a highly selective barrier, preventing the passage of toxic molecules and luminal bacterial translocation [24]. The normal barrier function is maintained by steady enterocyte turnover finely regulated by cell proliferation, migration, and apoptosis. Cell-to-cell contacts within the intestinal epithelium structured by a scaffold of tight and adherens junctions, located apically, are further responsible for sealing the intestinal barrier [25-27]. The results of this study suggest that selected antiretroviral drugs influence small intestinal absorptive and secretory functions.

We have assessed intestinal mucosal morphology, permeability changes, and electrolyte secretion following antiretroviral treatment in mice. All antiretroviral agents tested caused significant blunting of the absorptive villi, especially in the duodenum and jejunum. Moreover, hyperplasia of the crypt cells (which are responsible for the secretory function of the small intestine) were sometimes, noted in the duodenum (IDV, AZT) and jejunum (IDV). While HIV, per se, and concomitant enteric infections may cause mucosal atrophy [28,29], our findings suggest that antiretroviral drugs may also contribute to or aggravate any enteropathy secondary to infectious causes. Mannitol and lactulose assays have been very useful to assess overall intestinal absorptive area (mannitol) and impaired epithelial barrier function (lactulose), since both sugars are passively absorbed and excreted unchanged into the urine [19,30]. Interestingly, NFV was the only drug tested that caused significant increases in both lactulose and mannitol excretion rates, thus not affecting the lactulose:mannitol ratio. We speculate that NFV might have induced compensatory changes in villus area following intestinal epithelial injury. The other drugs tested did not show any significant change in lactulose excretion suggesting that epithelial tight junctions are not severely compromised by these other antiretroviral drugs. Bode et al has also described reductions in transepithelial electrical resistance (TEER) in HT-29/B6 intestinal cell monolayers, following exposure to NFV [7]. The effects of antiretroviral drugs on enterocyte tight junctions at the molecular level, however, remain to be explored. Interestingly, only AZT induced crypt hyperplasia in both the duodenum and jejunum compared with the other antiretroviral drugs (except IDV which only caused crypt hyperplasia in the duodenum). Proximal intestinal area reductions due to villus blunting and longer crypts cannot be excluded. In contrast, net sodium, chloride and water secretion are all significantly increased in animals treated with all drugs, except DDI, indicating that effects on electrolyte and water channels and thus, may help explain the development of diarrhea. It has been previously reported that NFV recruits a slowly inactivating basolateral Ca^2+ ^pathway, potentiating muscarinic Cl^- ^secretion, which may lead to secretory diarrhea. Interestingly, DDI did not cause an increase in Cl- or water transport, indicating a potentially different mechanism of action.

In addition to the side effects of PI-induced-diarrhea, HIV enteropathy and intestinal infections have also been shown to cause intestinal villus blunting and atrophy, leading to malabsorption [28,31]. We report in this study significant decreases in cell proliferation and increase in apoptotic and necrotic cell death *in vitro*, caused by NFV. Cell proliferation and death are crucial for the dynamic intestinal cell turnover process under normal physiologic conditions, but these responses become especially important during epithelial restitution following injury from various causes. Recently, it has been reported that NFV induces anchorage-independent growth and a G1 cell cycle arrest in melanoma cells by decreased activity of CDK2, a master kinase that regulates G1-S progression [32]. We postulate that DDI does not affect the cell cycle as does NFV, which would explain the lack of effects on cell proliferation *in vitro*. Previous studies have shown that PI's cause significant increase in apoptosis rates [7]. Recently, it has been shown that PI's activate the unfolded protein response (UPR), the ER specific stress response, disrupting the intestinal barrier integrity, and that reduction in CHOP expression, which is a UPR-induced transcription factor that mediates apoptosis, significantly reduced apoptosis both *in vitro *and *in vivo *[33]. These findings further help elucidate the different mechanisms by which HIV PI's can induce cell death in different cell lines. Indeed, our study showed a similar increase in cell apoptosis in groups treated with NFV both *in vivo *and *in vitro*. In addition, we also report a significant increase in necrosis rates caused by IDV and NFV, which had not been previously reported [7].

Our *in vitro *and *in vivo *models do not take into account other host and environmental variables including HIV infection itself or opportunistic intestinal pathogens that can certainly contribute to impaired intestinal function and responses to antiretroviral agents. HIV associated diarrhea may be partly related to muscarinic induced-chloride secretion [10]. Mucosal HIV replication might induce a pro-inflammatory and Th1-mediated cytokine over-expression [34], which could additionally disrupt the intestinal barrier with increased secretion with electrolyte and water loss. Depletion of lymphocytes in the lamina propria by HIV could disrupt the immune system and increase susceptibility to opportunistic infections [29,35]. Drug absorption may be further compromised by opportunistic enteric infections like *Cryptosporidium spp*. and Micromonospora spp., the former has been found in HIV-immunocompromised patients in Brazilian settings, with and without diarrhea [11,36,37]. Thus, a wide range of secretory intestinal transport disruption may occur *in vivo *due to direct antiretroviral therapy and both systemic and intestinal infections.

## Conclusion

In conclusion, this study focused on understanding the effects of protease inhibitors and reverse transcription antiretroviral drugs on the intestinal epithelium. Gaining insight into how antiretroviral agents cause diarrhea is essential in developing interventions that could potentially reduce these side effects, avoid therapy discontinuation and increase efficacy of the antiretroviral drugs. We have found that both PI's, NFV and IDV, decreased proliferation and increased necrosis *in vitro *and that only NFV increased apoptosis *in vitro*, in contrast to the reverse transcriptase inhibitors which did not have such an effect. Additionally, both PI's and reverse transcriptase inhibitors altered intestinal morphology *in vivo*, reducing villous height in the duodenum and jejunum and induced sodium secretion. Furthermore, NFV and IDV induced cell apoptosis *in vivo *and chloride and water secretion. NFV was the only drug tested that caused significant changes in intestinal permeability. Further studies are needed to elucidate molecular mechanisms involved in HIV PI-induced epithelial damage, including cell cycle studies, WNT-signaling and R-spondin-1, specific cell death pathways as well as studies to examine whether cell migration and cellular cytoskeletal F-actin are affected and contribute to disrupting the intestinal barrier. Additionally, these mechanisms might be modulated by important gut-trophic nutrients, such as glutamine, alanyl-glutamine, arginine and zinc [38], which could reduce intestinal damage, increase drug absorption and decrease undesirable side effects, such as diarrhea that can be a limiting factor for treatment adherence and efficacy.

## Competing interests

The authors declare that they have no competing interests.

## Authors' contributions

MB: Participated in the *in vitro *and *in vivo *experiments, performed data analysis and drafted the manuscript. CV: Participated in the *in vivo *experiments, data storage, analysis and helped drafting the manuscript. JG: Participated in the *in vivo *experiments and data analysis. JE: Participated in the *in vitro *experiments and data analysis. CA: Participated in the study design and writing the paper. RB: Participated in statistical analysis and drafting and reviewing of the manuscript. GB and BO: Participated in the *in vivo *experiments and critical reviewing. AL and RG: Participated in the coordination, conception, experiments design and critical reviewing and writing. All authors read and approved the final manuscript.

## Pre-publication history

The pre-publication history for this paper can be accessed here:

http://www.biomedcentral.com/1471-230X/10/90/prepub
